# Enhancing statin research for Alzheimer's prevention: Suggestions for future studies and policy implications

**DOI:** 10.1016/j.tjpad.2025.100118

**Published:** 2025-03-15

**Authors:** Yumei Zhong, Shanshan Liu, Xiaofeng Lv

**Affiliations:** a Chengdu Integrated TCM & Western Medicine Hospital/Chengdu First People's Hospital, Chengdu, PR China; b Sichuan Integrative Medicine Hospital, Chengdu, Chengdu, Sichuan, PR China; c Chengdu University of Traditional Chinese Medicine, School of Acupuncture and Tuina, Chengdu, PR China

To the Editor,

We read with great interest the article “*Statin Use and Alzheimer's Disease Risk: The Impact of Genetic and Individual Factors*,” which provides valuable insights into the role of statins in Alzheimer's disease (AD) prevention^1^. The study sheds light on the potential benefits and risks of statins in at-risk populations. However, we would like to make some constructive suggestions for the future.

First, the study associates statin use with AD risk but does not fully address reverse causality. AD may lead to increased statin prescriptions due to comorbidities such as cardiovascular disease, rather than statins causing AD^2^. Future research should utilize Mendelian randomization to better establish causality and use prospective cohort designs with baseline cognitive assessments to reduce bias. Second, the study primarily uses global cognitive scores without examining specific cognitive domains, such as episodic memory or executive function, which are more sensitive to AD^3^. Longitudinal assessments and more detailed cognitive tests would provide a clearer picture of statin effects. Additionally, clarifying assessment protocols (e.g., blinding and examiner consistency) would minimize bias. Third, the study does not account for competing risks such as stroke, diabetes, or cardiovascular disease, which could confound the relationship between statin use and AD^4^. Future research could adopt competing risks models, like Fine-Gray models, to adjust for the influence of other health conditions and provide more accurate estimates of statin effects on AD.

In addition, we believe that some reflections may be crucial for public health policy. In the first place, given the public health implications of Alzheimer's disease, especially in an aging global population, the findings of this study underline the need for policy changes that promote personalized treatment strategies. We advocate for the integration of genetic screening into routine healthcare practices to identify individuals who may benefit most from statin therapy in the context of AD prevention. This could lead to more targeted use of statins, helping to reduce the overall burden of AD in high-risk populations. Furthermore, public health initiatives should emphasize early cognitive screening for at-risk individuals, particularly those who are older or have a family history of dementia, in order to detect early cognitive decline before it progresses to Alzheimer's.

In conclusion, while the study provides valuable insights, addressing reverse causality, improving cognitive assessments, and incorporating competing risks models will strengthen future research on statins and AD prevention. We appreciate the authors’ work and look forward to further studies in this critical area.

## Financial support

This research received no external funding.

## Declarations of AI use

We have not used any AI at all.

## CRediT authorship contribution statement

**Yumei Zhong:** Data curation, Methodology, Writing – original draft. **Shanshan Liu:** Methodology, Writing – original draft, Writing – review & editing. **Xiaofeng Lv:** Methodology, Supervision, Writing – original draft, Writing – review & editing.

## Declaration of competing interest

The authors declare that they have no known competing financial interests or personal relationships that could have appeared to influence the work reported in this paper.
